# Synthesis of Crocin I and Crocin II by Multigene Stacking in *Nicotiana benthamiana*

**DOI:** 10.3390/ijms241814139

**Published:** 2023-09-15

**Authors:** Lei Xie, Zuliang Luo, Xunli Jia, Changming Mo, Xiyang Huang, Yaran Suo, Shengrong Cui, Yimei Zang, Jingjing Liao, Xiaojun Ma

**Affiliations:** 1 Institute of Medicinal Plant Development, Chinese Academy of Medical Sciences, Peking Union Medical College, Beijing 100193, China; leixie1996@163.com (L.X.); zuliangluo@163.com (Z.L.); xunlijia1998@163.com (X.J.); c1061729635@163.com (S.C.); meiyee0810@sina.com (Y.Z.); 2 Guangxi Crop Genetic Improvement and Biotechnology Lab, Guangxi Academy of Agricultural Science, Nanning 530007, China; mochming@126.com; 3 Guangxi Key Laboratory of Plant Functional Phytochemicals and Sustainable Utilization, Guangxi Institute of Botany, Guangxi Zhuang Autonomous Region and Chinese Academy of Sciences, Guilin 541006, China; xiyangHuangGxib@126.com; 4 State Key Laboratory for Quality Ensurance and Sustainable Use of Dao-di Herbs, Artemisinin Research Center, and Institute of Chinese Materia Medica, China Academy of Chinese Medical Sciences, Beijing 100700, China; suoyaran213@163.com

**Keywords:** crocins, crocin I, crocin II, multigene assembly, plant chassis, synthetic biology

## Abstract

Crocins are a group of highly valuable water-soluble carotenoids that are reported to have many pharmacological activities, such as anticancer properties, and the potential for treating neurodegenerative diseases including Alzheimer’s disease. Crocins are mainly biosynthesized in the stigmas of food–medicine herbs *Crocus sativus* L. and *Gardenia jasminoides* fruits. The distribution is narrow in nature and deficient in resources, which are scarce and expensive. Recently, the synthesis of metabolites in the heterologous host has opened up the potential for large-scale and sustainable production of crocins, especially for the main active compounds crocin I and crocin II. In this study, *GjCCD4a*, *GjALDH2C3*, *GjUGT74F8*, and *GjUGT94E13* from *G. jasminoides* fruits were expressed in *Nicotiana benthamiana*. The highest total content of crocins in T1 generation tobacco can reach 78,362 ng/g FW (fresh weight) and the dry weight is expected to reach 1,058,945 ng/g DW (dry weight). Surprisingly, the primary effective constituents crocin I and crocin II can account for 99% of the total crocins in transgenic plants. The strategy mentioned here provides an alternative platform for the scale-up production of crocin I and crocin II in tobacco.

## 1. Introduction

Crocins are highly valuable water-soluble carotenoids that were first discovered in the dried stigmas of the valuable food–medicine and traditional Chinese medicine, saffron. Saffron has distinct pharmacological actions such as blood-activation and resolving stagnation for tranquilization [1]. Moreover, due to its unique aroma and bright color, saffron is often used as a natural colorant and spice [2]. Consequently, as the main component of the saffron pigment, crocins are often considered for the treatment of Alzheimer’s disease [3] and various liver diseases, including chemical liver injury, liver fibrosis, liver cancer, etc. [4,5,6]. Crocins are classified into five types: crocin I, crocin II, crocin III, crocin IV, and crocin V, based on different numbers of glucosyl. Crocin I has four glucosyls, crocin II has three, crocin III and crocin IV have two, and crocin V has one [7]. Among these, crocin I and crocin II are the main components and have even been considered the main active ingredients, serving as quality indicators for saffron quality evaluation. *Chinese Pharmacopoeia* stipulates that the content of crocin I and crocin II in saffron medicinal materials must not be less than 10% [1]. All pharmaceutical companies in China need to comply with this regulation. Modern pharmacological research has further shown that crocin I and II have memory-improving [8], radiation-protection [9], antidepressant [10], anticancer [11], and antioxidant effects [12], thereby demonstrating great potential for clinical application and advantages in new drug development, food, and cosmetics industries. However, saffron is commonly referred to as “red gold”, due to its lower yield and higher price [13]. Currently, crocins are primarily isolated from saffron; however, the limited sources, low yield, complex extraction process, and lack of environmental friendliness severely restrict their market applications. Therefore, there is an urgent need to develop a green and efficient strategy for crocin production.

**Figure 1:**
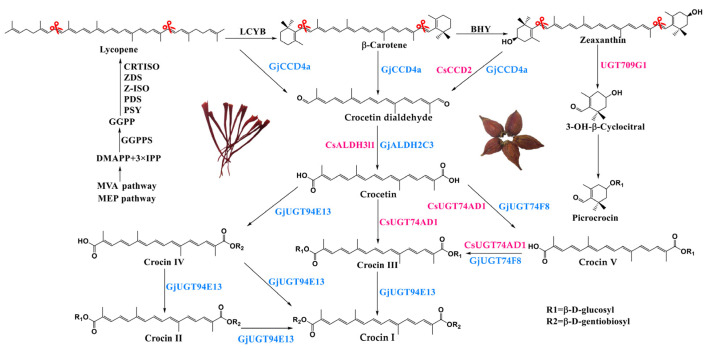
The crocin biosynthetic pathway in *Crocus sativus* L. (pink) and *Gardenia jasminoides* (blue). PSY, phytoene synthase; PDS, phytoene desaturase; Z-ISO, ζ-carotene isomerase; ZDS, ζ-carotene desaturase; CRTISO, carotenoid isomerase; LCYB, lycopene β-cyclase; BHY, β-carotene hydrolase; GjCCD4a and CsCCD2, carotenoid cleavage dioxygenase; GjALDH and CsALDH3l1, aldehyde dehydrogenase; GjUGT74F8, GjUGT94E13, CsUGT74AD1, and UGT709G1, UDP-glucosyltransferase.

Over the years, the biosynthetic pathway of crocin in *Crocus sativus* L. and *Gardenia jasminoides* has been elucidated (Figure 1) [14,15,16]. Three molecules of isopentenyl diphosphate (IPP) react with dimethylallyl diphosphate (DMAPP), which are derived from the mevalonic acid (MVA) pathway or the meth-ylerythritol 4-phosphate (MEP) pathway to generate geranyl-geranyl diphosphate (GGPP) under the action of geranylgeranyl pyrophosphate synthase (GGPPS) [17]. GGPP undergoes a series of processes involving enzymes such as phytoene synthase (PSY), phytoene desaturase (PDS), ζ-Carotene Isomerase (Z-ISO), ζ-Carotene desaturases (ZDS), and carotenoid isoenzymes (CRTISO) to produce lycopene. Subsequently, lycopene is converted into β-carotenoids via the action of lycopene β-cyclinase (LCYB). Further catalysis via β-Carotene hydrolase (BHY) leads to the production of zeaxanthin. Lycopene, β-carotene, and zeaxanthin are further cleaved by carotenoid cleavage dioxygenases (CCDs) and converted into crocetin dialdehyde. Then, aldehyde dehydrogenases (ALDHs) catalyze the conversion of crocetin dialdehyde into crocetin and finally, glycosylated by UDP-glucosyltransferases (UGTs) to produce five crocins [18,19]. In addition, the pyrolytic product of zeaxanthin is oxidized to 3-OH-β-Cyclocitral and then glycosylated by UGT709G1 to produce picrocrocine [20]. 

Since the crocin biosynthetic pathway of crocins was elucidated, people have been concerned about the heterologous expression of crocin, leading to the transfer of relate genes into microbial chassis due to its many activities. Chai et al. successfully introduced *PsCrtZ*, *CsCCD2*, and *ScaLD* genes into *Saccharomyces cerevisiae*, resulting in a yield of 6278 μg/L of crocetin via fermentation [21]. Wang et al. synthesized crocetin de novo in *Escherichia coli* with a content of 4.42 mg/L [22]. Pu et al. transformed *GjUGT74F8* and *GjUGT94E13* into *E. coli* with substrate crocetin feeding, achieving a conversion rate of 66.1% [23]. The problem of high energy consumption in microbial fermentation and plant chassis with the potential for large-scale agricultural production, cost-effectiveness and environmental concerns has long been underestimated. 

Currently, crocins have been successfully expressed in potatoes and tomatoes, with the main product being crocin II [24,25,26]. Gómez-Gómez et al. used the plants’ own glycosyltransferase to produce crocins in *Nicotiana* species via a series of transformation combinations of *CsCCD2L* and other genes related to carotenoid precursor synthesis and achieved a content of almost 400 µg/g DW (dry weight) in leaves [27,28]. However, the main active ingredients crocin I and crocin II only account for about 40%, which needs further improvement. Salim Al-Babili et al. overexpressed *GjCCD4a* in tobacco, and the product was also mainly crocin II [29]. In an article published in June of this year, Giovanni Giuliano et al. used viral vectors to transiently express *CsCCD2* in genome-edited plants with the highest β-carotene and zeaxanthin to produce a high proportion of crocin I and crocin II, of course mainly crocin II, leading to a total crocins content of up to 800 μg/g DW [30].

Glycosylation can improve the hydrophilicity and pharmacokinetics of lipophilic compounds [23]. At present, the main component in transgenic tobacco is crocin II and the proportion of crocin I is not too high. Additionally, biomass and production costs need to be taken into account if tobacco is to be used as a source for crocin extraction. It is clear that there is still some room for improvement in current research. To address this, we adopted the strategy of in-fusion and 2A polypeptides to build a polygenic vector with four genes including *GjCCD4a*, *GjALDH2C3*, and two glucosyltransferases genes *GjUGT74F8* and *GjUGT94E13* to produce crocins in *Nicotiana benthamiana*. We hope to establish a new mode of plant chassis production of crocin by transferring AU-CU vectors with more types of substrates to model species of tobacco. Moving forward, we will further optimize the multi-gene expression vector and then introduce it into other potential plant chassis to serve as sources for crocin production to confirm the most suitable plant chassis.

## 2. Results

### 2.1. Multigene Expression Vector Construction

**Figure 2:**
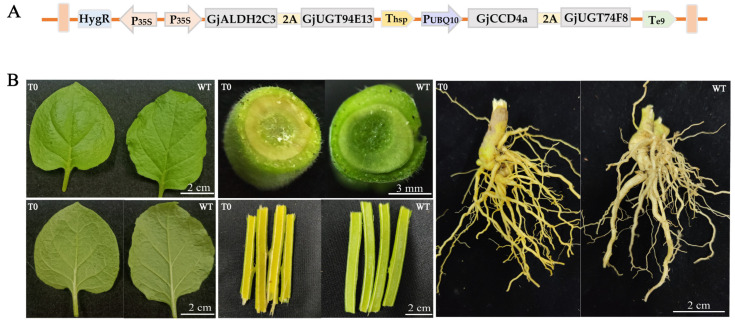
Genetic transformation of AU-CU vector plants of T0 generation. (**A**). T-DNA region of the multigene vector AU-CU that was transformed into the tobacco plants. (**B**). Compare the leaves, roots, and stems of AU-CU transgenic plants with those of the wild-type.

In this study, we synthesized four genes involved in the crocins biosynthesis, including carotenoid cleavage dioxygenase gene *GjCCD4a*, aldehyde dehydrogenase gene *GjALDH2C3*, two UDP-glucosyltransferase genes *GjUGT74F8* and *GjUGT94E13*, and then constructed a multigene expression vector for crocin production in tobacco. We first built two double-gene expression vectors; *GjCCD4a* and *GjUGT74F8* were linked by 2A peptides and inserted into pBI121 with the AtUBQ10 promoter and E9 terminator. In addition, *GjALDH2C3* and *GjUGT94E13* were connected with 2A peptides and inserted into pBI121 with CaMV 35S promoter and heat-shock protein (HSP) 18.2 terminators. Then, the two double-gene vectors were used as templates, and the promoters, target genes, and terminators were cloned and fused together into the pCAMBIA1300 vector, resulting in the construction of the multigene expression vector named AU-CU. The structure of the vector was as follows: 35s-GjALDH2C3-2A-GjUGT9F13-Thsp-UBQ-GjCCD4a-2A-GjUGT74F8-TE9 (Figure 2A and Figure S2). 

We obtained the recombinant plasmid by sequencing identification and PCR amplification. The AU-CU vectors were transformed into *Nicotiana benthamiana* by *Agrobacterium tumefaciens* to synthesize five crocins (Figure S1). Five transgenic plants N16, N18, N20, N22, and N34 lines were obtained via screening with hygromycin. Comparing the appearance characteristics of plants’ roots, stems, and leaves, there were significant differences between the transgenic plants and the wild type (Figure 2B). Due to the influence of pigments such as chlorophyll, the color was not obvious under the normal lens. However, it was evident that the epidermis of the transgenic tobacco stems appeared more yellow, especially the transverse and longitudinal sections of the stems, and the roots had more significant color differences with little or no color interference.

### 2.2. Genetic Transformation of Nicotiana benthamiana and Molecular Identification

**Figure 3:**
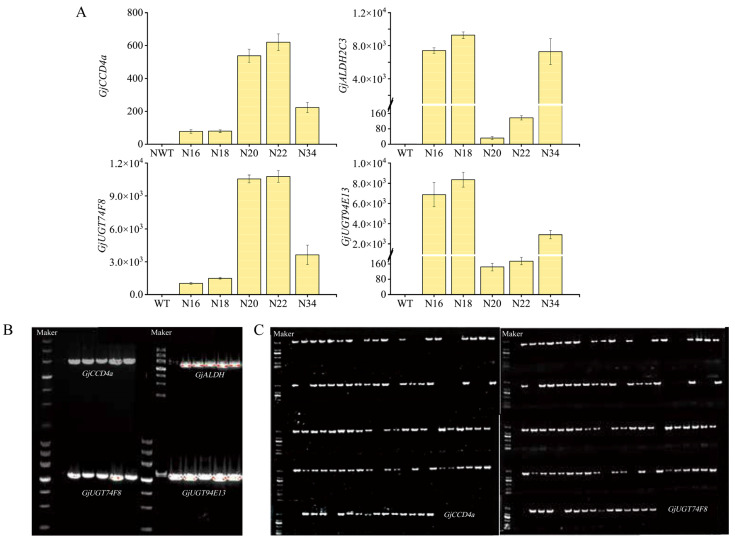
Genetic Transformation of *Nicotiana benthamiana* with multigene vector synthesizing crocins and molecular identification. (**A**) The relative gene expression level analysis of crocins synthesis genes in transgenic tobacco lines. The Nbactin was used as an internal control and the wild-type was set to 1. The data are presented as the mean values ± SDs, *n* = 3 biologically independent samples. (**B**) PCR detection of crocins biosynthetic genes. The first lane is the DNA maker (4500 bp), the second is the wild type, and then from left to right are transgenic *Nicotiana benthamiana* lines N16, N18, N20, N22, and N34. (**C**) PCR to identify whether the T1 generation carries *SgCCD4a* and *SgUGT74F8*. The first lane of each row is the DNA maker (2000 bp), the second is the wild type, and the rest are the T1 generations.

In order to evaluate the possibility of using this type of tobacco to synthesize crocins, we identified whether the gene was integrated into the tobacco genome, and extracted the leaves DNA of resistant plants and wild-type (WT) tobacco. Four genes, namely *GjCCD4a*, *GjALDH2C3*, *GjUGT74F8*, and *GjUGT94E13* were amplified using specific primers and the lengths of the four genes were 1995 bp, 1512 bp, 1341 bp, and 1374 bp, respectively (Figure 3B). Four genes in the leaves of the T0 generation plant were identified using qPCR. Compared to wild-type tobacco, it was observed that the expression levels of these genes in the transgenic tobacco were significantly higher, indicating the successful expression of the synthetic crocin genes. Notably, the N20 line exhibited the highest expression level of *GjCCD4a*, which was eight times higher than that of the N16 line. The N18 line showed the highest expression level of *GjALDH2C3*. The N20 and N22 lines had higher expression levels of *GjUGT74F8* compared to the N16, N18, and N34 lines. On the other hand, the N16 and N18 lines exhibited higher expression levels of *GjUGT94E13* than the N20, N22, and N34 lines, particularly the N20 and N22 lines (Figure 3A). Gene loss is a common issue in multigene transgenic plants. To study the genetic stability of the plant after being transferred into the multigene vector, we sowed T1 seeds of transgenic tobacco and randomly selected 108 tobacco plants for genomic DNA extraction and PCR identification of *GjCCD4a* and *GjUGT74F8* with nonspecific bands. There were 22 non-positive plants, and the rate was about 20.4% (Figure 3C).

### 2.3. Identification and HPLC-MS/MS Analysis for Crocins of T0 Generation

**Figure 4:**
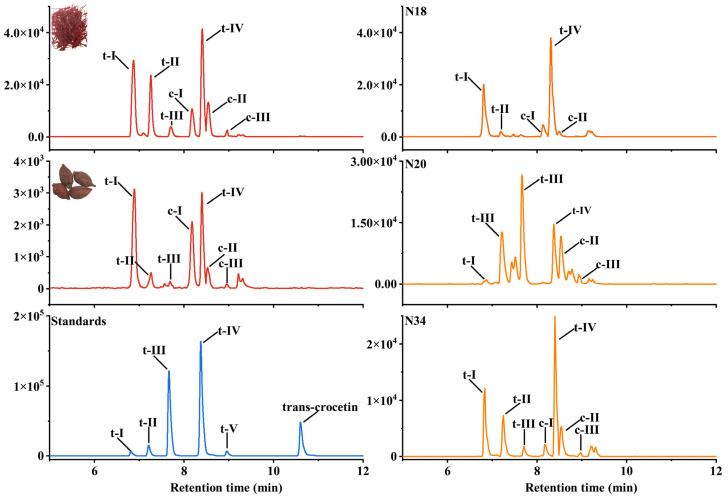
Comparison of the production of crocins crocetin between *Crocus sativus* L. stigmas, *Gardenia jasminoides* fruits, and transgenic tobacco. The red curve represents HPLC-MS/MS analysis of crocins in *Crocus sativus* L. stigmas and *Gardenia jasminoides* fruits. The blue curve represents HPLC-MS/MS analysis of crocins and crocetin standards. The orange curve represents HPLC-MS/MS analysis of crocins in N18, N20, and N34 lines; t-I, c-I, t-II, c-II, t-III, c-III, t-IV, and t-V represent trans-crocin I, cis-crocin I, trans-crocin II, cis-crocin II, trans-crocin III, cis-crocin III, trans-crocin IV and trans-crocin V, respectively.

Based on molecular analysis, to prove that the multigene vector effectively expresses the genes, the composition and content of crocins in the leaves of transgenic tobacco lines were determined using HPLC-ESI-MS/MS. Trans-crocetin trans-crocin V, trans-crocin IV, trans-crocin III, trans-crocin II, and trans-crocin I standards were detected at retention times of 10.60, 8.95, 8.37, 7.67, 7.21, and 6.82 min (Figure 4). According to the characteristic ions and combined with the retention time of ion pairs extracted from saffron and *G. jasminoides* fruits, we confirmed the retention times of the cis-trans configuration in the transgenic tobacco leaves, cis-crocin III cis-crocin II, cis-crocin I in 8.91, 8.48, 8.19 min, respectively (Figure 4). The content of crocins in the N18 line was found to be the highest at 22,811 ng/g FW (fresh weight). Furthermore, it was observed that after transferring the AU-CU vector with downstream glycosyltransferase genes *GjUGT94E13* and *GjUGT74F8*, the substrate crocetin was efficiently consumed, resulting in extremely low levels of crocin V. The N16 and N18 transgenic lines primarily accumulated crocin I, with crocin I accounting for up to 89% in the N18 line and 88% in the N16 line. On the other hand, the N20 and N22 transgenic lines mainly accumulated crocin II, with the N22 line containing up to 77% crocin II and the N20 line containing 66% crocin II. The proportion of crocin I and crocin II in the N34 transgenic line was 64% and 28%, respectively (Figure S3). In addition, it can be noted that the ratio of crocin I and crocin II in the N34 line was the closest to that of saffron and *G. jasminoides* fruits in all transgenic tobacco plants (Figure 4). 

In addition to HPLC-ESI-MS/MS analysis, we conducted further metabolite identification using UPLC-ESI-QTOF-MS/MS. By comparing the retention time, molecular weight, and mass spectrometry analysis of standards, we identified the presence of cis-trans crocin I, II, III, trans-crocin IV, and trans-crocin V in the transgenic tobacco plants. In the MS/MS spectrum, [M + Na]^+^ ions were commonly observed in positive ion mode. Additionally, [M + H]^+^, [M + K]^+^, and [M + NH4]^+^ ions were also detected, although their abundance was relatively low (Figures S6–S11). Furthermore, we detected cis-trans crocetin and cis-crocin V in the stems of the N20 line (Figure S4).

### 2.4. HPLC-MS/MS Analysis for Crocins of T1 Generation

**Figure 5:**
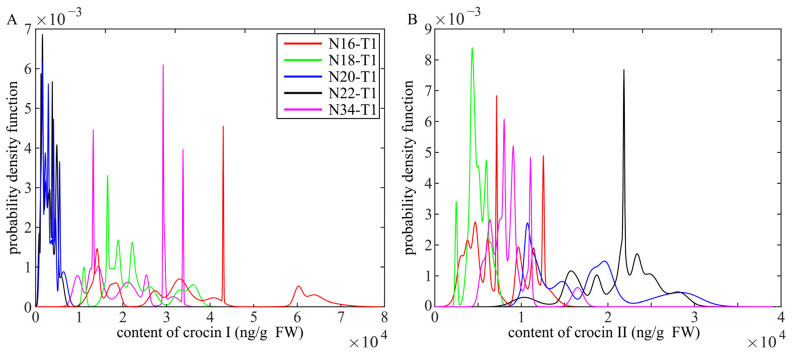
Probability density distribution curve of T1 generation crocin I and crocin II. (**A**). Probability density distribution curve of crocin I. (**B**). Probability density distribution curve of crocin II. The red curve represents the T1 generations of the N16 line; the green represents the T1 generations of the N18 lines; and the blue represents the T1 generations of the N20 line. The black represents the T1 generations of the N22 line and the pink represents the T1 generations of the N34 line. The curve plotting data are the mean values ± SDs, *n* = 3 biologically independent samples.

Transgenic plants are mostly heterozygotes, and character segregation often occurs in the T1 generations. We selected 15 transgenic plants with obvious appearance traits from each line for content testing, which were confirmed via PCR. The T1 generation lines with the highest content of each T0 transgenic tobacco line were designated as N16-HT1, N18-HT1, N20-HT1, N22-HT1, and N34-HT1. The probability density distribution curve of crocin I in the T1 generations showed that the N16-T1 line had the largest moving distance to the right, followed by the N18-T1 and N34-T1 lines. The N20-T1 and N22-T1 lines predominantly concentrated within the range of 800–6500 ng/g FW. On the other hand, in the probability density distribution curve of crocin II, the curves of the N20-T1 and N22-T1 lines moved the furthest to the right and were mainly concentrated in the range of 20,000–27,000 ng/g FW. The probability curve for the N34−T1 line had a relatively central distribution range in both graphs. These results indicate evident and complex character differentiation in the T1 generations. The content of crocin I in the N16-T1 and N18-T1 lines remained significantly higher than that in the N20-T1 and N22-T1 lines. Conversely, the crocin II content in the N20-T1 and N22-T1 lines was generally higher compared to the N16-T1 and N18-T1 lines (Figure 5A,B and Figure S5).

**Figure 6:**
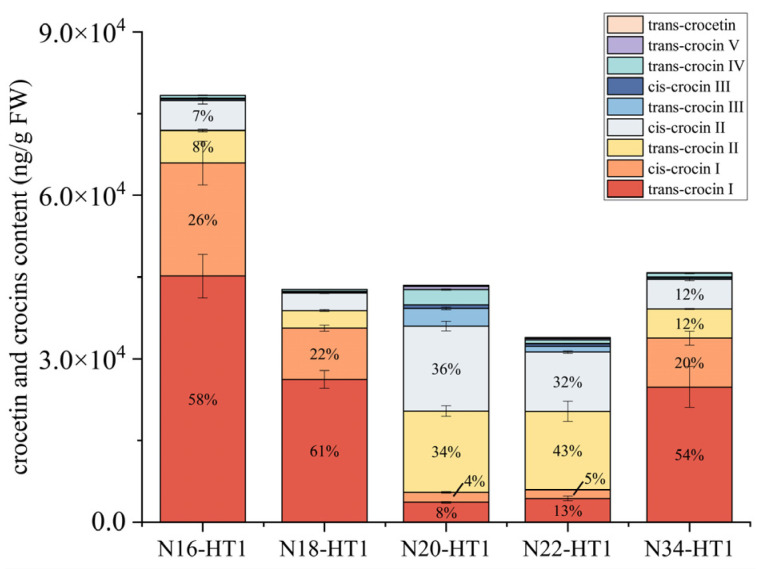
Accumulation of crocins in transgenic tobacco lines of T1 generation. The data are presented as the mean values ± SDs, *n* = 3 biologically independent samples.

In the T1 generation, the plants exhibited higher crocin content compared to the T0 generation. The plants with the highest crocin content in the T1 generation had approximately 1–3 times higher levels than those in the T0 generations. The total crocins content of N16-HT1, N18-HT1, N20-HT1, N22-HT1, and N34-HT1 lines was 78,362 ng/g FW, 42,705 ng/g FW, 43,271 ng/g FW, 33,700 ng/g FW, and 45,796 ng/g FW, respectively. Furthermore, trans-crocetin was only detected in the N20-HT1 and N22-T1 lines, with levels of 191 ng/g and 181 ng/g FW, respectively. The N16-HT1 line had the highest content of crocin I, reaching 65,953 ng/g FW, which accounted for 84% of crocin biosynthesis. This proportion was the highest among the reported studies on crocin biosynthesis. Crocin I and crocin II together comprised 99% of the crocins in the N16-HT1 line. The content of crocin II in N20-HT1 was 30,507 ng/g FW, accounting for 70% of the crocins and crocetin content. Notably, the content of crocin I in the N34-HT1 line was three times higher than that of crocin II (Figure 6). 

This study has significantly increased the proportion of the main components crocin I and crocin II in transgenic tobacco. This suggests that the gene composition of the multi-gene vector used in the study is relatively well-suited for this purpose. The findings provide a research basis for subsequent vector modification and the search for better plant chassis as alternatives to saffron for extraction. Additionally, the expression levels of GjUGT74F8 and GjUGT94E13 have been found to exert a significant impact on the production of crocin I and crocin II. In the future, the transfer of only one glycosyltransferase may suffice to achieve higher levels of monomer expression and reduce the cost of acquiring monomers via separation.

### 2.5. Determination of Moisture Content 

It is often necessary to dry plant samples in the production process, especially for the purpose of extraction. In order to estimate the dry weight of genetically modified to know the productive potential, we need to know the moisture content of the tobacco leaves. The moisture content was measured by the drying method: 1–2 g of transgenic tobacco leaves were taken and dried in a weighing bottle with a constant weight under 105 °C for 6 h, transferred to a dryer to cool for 30 min, precisely weighed, dried at the same temperature for 1 h, cooled, and weighed until the difference between two consecutive times was no more than 5 mg and conducted four parallel experiments. The moisture content of transgenic tobacco leaves was calculated, and the measured moisture content was 92.5%, 92.5%, 92.2%, 92.3%, and the average value was 92.4%. Using this moisture content value, it can be estimated that the crocin content in the T1 generation transgenic tobacco can reach 1,058,945 μg/g DW.

## 3. Discussion

Via the strategy of fusion combined with the 2A polypeptide connection that was used in previous studies, we have successfully constructed a multi-gene vector containing four genes. These genes were effectively expressed in tobacco plants for crocin synthesis, resulting in the production of crocins with a maximum content of 78,362 ng/g FW. In previous studies, glycosyltransferase and ALDH were not introduced into tobacco [24,27,28], so the potential has not been fully understood in crocin biosynthesis. Via the analysis of experimental results, we further optimized the AU-CU carrier. Additionally, it is our hope that this study can serve as a valuable reference for future research on crocins biosynthesis in plant chassis.

It should be noted that in this study, we transformed two downstream glycosyltransferase genes *GjUGT74F8* and *GjUGT94E13*, achieving higher substrate conversion efficiency that solved the problem of low proportion of the main active components crocin I and crocin II, especially crocin I in previous research for synthesizing crocin in transgenic tobacco [24,27,28]. We only detected the presence of crocetin in a few lines, and the content of crocin V, IV, and III was also very low, while the proportion of crocin I and crocin II can reach a maximum of 99%, and the highest proportion of crocin I can reach up to 84%. In addition, the proportion of the N34 line was relatively similar to that of saffron and *G. jasminoides* fruits (Figure 4). Visibly, the ratio of crocin I to crocin II was related to the expression levels of *GjUGT74F8* and *GjUGT94E13*. The lines with high expression levels of *GjUGT74F8* had a higher content of crocin II, such as the N20 and N22 line, while the lines with high expression levels of *GjUGT94E13* had more crocin I, for example, the N16 and N18 lines (Figure 3). The main active components crocin I and II were the main components, just like its original plant, which indicated the necessity of converting downstream glycosyltransferases. It is not difficult to conclude that by transferring only *GjUGT74F8* or *GjUGT94E13*, a higher proportion of crocin I or II can be obtained, which is very beneficial for the extraction and purification of monomers. The ratio of crocins in the N34 line was very similar to that of saffron and *Fructus Gardeniae*, which means that when the expression level of each gene in the synthesis pathway is appropriate, it is possible that the proportion of metabolites is also very close to that of the original plant. This is of great significance for the food fortification of crops and ensures food safety to a certain extent. 

With the exception of glycosyltransferases, aldehyde dehydrogenases (ALDH) were used in this study. The ALDH family is widely distributed in plants and can oxidize aldehyde oxides to corresponding carboxylic acid compounds. However, ALDH exhibits strong specificity in catalyzing crocetin dialdehyde; Demurtas et al. screened six *CsALDH* genes, and in the end, only CsALDH3I1 was able to catalyze the production of crocetin dialdehyde to crocetin [31]. Although exogenous ALDHs are not required for the synthesis of crocins in tobacco, tomato, and potato, it does not mean that all plants have this catalytic function. As an important step in the synthesis pathway, the auxiliary role of ALDH was also very important in this study. Although the expression level of *GjCCD4a* is low in the N16 and N18 lines, the final crocins accumulation was higher than in the N20 and N22 lines. From the expression level of genes, ALDH may be the main reason because their expression levels of *GjALDH2C3* are much higher than the N20 and N22 lines, with a difference of nearly 300 times. By transferring exogenous genes to enhance the enzyme activity of certain steps, the content of the end products can be increased, which is equivalent to increasing the copy number of the enzyme gene. Gómez-Gómez et al. t transferred vector with *CsCCD2L*, β-carotene hydroxylase gene (*BrCrtZ*) and the Arabidopsis thaliana ORANGE mutant gene (*AtOrMut*) into tobacco to produce crocin [28]. Therefore, although enzymes with the same function already exist in transgenic plants, reasonable design and assembly will also become the key to increasing their content. ALDH, as an important enzyme for the accumulation of substrate crocetin, and its application and efficiency screening is of great concern for the future heterologous biosynthesis of crocins. In this study, the *GjCCD4a* we used was derived from *G. jasminoides.* According to previous studies, carotenoid cleavage dioxygenases from *Bixa orellana, B. davidii, C. ancyrensis,* and *Crocus sativus* have the function of cleaving carotenoids to produce crocetin dialdehyde [16]. Lycopene, β-carotene, and zeaxanthin are the main substrates of CCDs, but different CCDs have different substrate preferences. For example, GjCCD4a and BoCCD4-3 can cleave lycopene, β-carotene, and zeaxanthin, while CsCCD2 and BdCCD4 prefer to use zeaxanthin as substrates [24,32,33]. However, according to the research of others, the activities of different CCDs are various, and aldehyde dehydrogenases (ALDHs) are widely found in plants. There are many ALDHs that catalyze the formation of crocetin from crocetin dialdehyde [34,35,36] but their activity has not been compared sufficiently. We can choose more efficient CCDs and ALDHs or directed evolution to optimize the multigene vector to product crocins in the future.

At present, there have been many studies on the heterologous expression of saffron in plants; however, applying it to the actual extraction of crocins is still a problem. To replace saffron as an extraction material, the content of crocins needs to be high first, and secondly, the growth rate and biomass of the chassis plant are also important. It is important to choose a suitable plant chassis, occupy less land resources, and obtain more extraction materials. Tobacco is a good chassis plant, which grows fast and has a large biomass. However, there are many kinds of fast-growing plants with high biomass. It is necessary to find a more suitable chassis or modify the plant chassis, such as gene-editing plants with more carotenoids such as transgenic materials.

In addition, the optimization of carriers is also essential. Firstly, the type of carrier and the gene composition of the carrier can also be assembled more reasonably. On one hand, it is necessary to select genes with higher activity or to increase the copy number of key genes. On the other hand, a reasonable combination of genes is crucial. Carotenoids and chlorophyll have a competitive substrate relationship, which may affect chlorophyll synthesis and then affect plant growth while reducing downstream conversion efficiency. In addition, the generation of crocetin consumes carotenoid substrates, which are protective factors for photosynthesis. The excessive production of crocins may affect the normal physiological state of the plant. So, we will also consider adding the rate-limiting enzyme HMGR of the MVA pathway and GGPPS, which plays an important role in carotenoid and chlorophyll synthesis. In previous reports, the rational modification of GGPPS can significantly increase the content of carotenoids and chlorophyll in tobacco [37].

After successful exogenous gene transfer, gene loss is normal. T-DNA insertion into the nuclear genome is random and the T0 generations are often heterozygous; the genes are easily lost during offspring separation due to homologous chromosome pairing and exchange. Via the PCR identification of T1 generations, the non-positive plants are close to 1/4, and no noticeable gene loss. However, because T0 may have chimeras and the chromosome positions inserted by each line may be different, it is necessary to obtain a homozygote and self-pollinate to determine whether gene loss exists. In the content determination of the T1 generation, we found evident character segregation. We selected the plant line with the highest content and will investigate whether there is any gene loss based on the trait segregation and the number of negative plants in the T2 generations.

## 4. Materials and Methods

### 4.1. Plant Material, Chemicals, and Strains

*Nicotiana benthamiana* was used for genetic transformation, the seeds of this tobacco from Laboratory stocks. *Escherichia coli* strains DH5α and XL10-Gold, and *Agrobacterium tumefaciens* strain GV3101 (WeidiBio, Shanghai, China) were used in this study. HPLC-grade acetonitrile, methanol, and formic acid were purchased from Fisher (Emerson, IA, USA). The ClonExpress Ultra One Step Cloning Kit, ClonExpress MultiS One Step Cloning Kit, ClonExpress Ultra One Step Cloning Kit, 2× Rapid Taq Master Mix, HiScript III 1st Strand cDNA Synthesis Kit (+gDNA wiper), and Taq Pro Universal SYBR qPCR Master Mix were purchased from Vazyme Biotech Co., Ltd. (Nanjing, China). The Ultrapure RNA Kit (DNase I) was obtained from CWBIO. Co., Ltd. (Beijing, China). The plant genomic DNA kits, restriction enzymes, and DNA Marker III were purchased from TianGen Biotech Co., Ltd. (Beijing, China). The KOD OnePCR master Mix was obtained from TOYOBO Biotech Co., Ltd. (Shanghai, China). The PBI121 and pCAMBIA1300 plasmids were from laboratory stock. The baicalein standard was obtained from Shanghai Yuanye Bio-Technology Co., Ltd. (Shanghai, China) unless otherwise specified. All crocin standards, including crocin V, crocin IV, crocin III, crocin II, and crocin I, as well as crocetin were purchased from the third-party scientific research platform CASmart (https://www.casmart.com.cn/ [1 January 2023]).

### 4.2. Multigene Expression Vector Construction

According to the gene sequence provided by NCBI, *GjCCD4a* (KY631925), *GjALDH2C3* (KY631926), *GjUGT74F8* (MN944054) and *GjUGT94E13* (MN944055) for the synthesis of crocins in *Gardenia jasminoides* were chemically synthesized via Sangon (Shanghai, China). The used promoter AtUBQ10 was cloned from the *Arabidopsis thaliana* genome using KOD One PCR master Mix, the CaMV35s promoter was amplified from PBI121 plasmid, and the pea rbcS-E9 gene terminator (Te9) and heat shock protein (HSP) 18.2 terminator were chemically synthesized using GENEWIZ (Suzhou, China). *GjALDH2C3* and *GjUGT94E13* were ligated into the PBI121 vector with CaMV 35S promoter and heat-shock protein (HSP) 18.2 terminators at the *BamHI/SacI* site via 2A linker peptide (the amino acid sequence is GSGATNFSLLKQAGDVEENPGP). In the same way, *GjCCD4a* and *GjUGT74F8* were linked by 2A peptide to the PBI121 vector with the AtUBQ10 promoter and E9 terminator. 35S-GjALDH2C3-2A-GjUGT94E13-Thsp and AtUBQ10-GjCCD4a-2A-GjUGT74F8-TE9 were amplified from PBI12 and connected to the pCAMBIA1300 vector at the *ECORI/HindIII* site, then formed a polygenic vector AU-CU of synthesizing a variety of crocins. Finally, the AU-CU vectors were transformed into tobacco via *Agrobacterium tumefaciens* strain GV3101.

### 4.3. Nicotiana benthamiana Transformation

Approximately 100 tobacco seeds were disinfected with 10% NaClO at 10 min and 75% ethanol at 30 s, then sown in 1/2 MS medium, cultured for more than one month after tobacco germination, using the leaf disc method to finish the genetic transformation. The tobacco leaves were cut into about 1 cm3, pre-cultured at 25 °C for 2 d in the dark (MS with 1 mg/L 6-BA, 0.2 mg/L NAA); approximately 500 leaves were cut, then infected the leaf explants with the strain GV3101 carrying the polygene vector for 15 min, and cocultured in the dark at 25 °C for 3 d. The explants were regenerated on a specific medium (MS with 1 mg/L 6-BA, 0.2 mg/L NAA, 400 mg/L cefotaxime, and 7 mg/L hygromycin) under a 16 h/8 h (light/dark) photoperiod at 25 °C. When the buds grew to about 2–3 cm, they were transferred to the rooting medium (MS with 0.2 mg/L NAA, 400 mg cefotaxime and 7 mg/L hygromycin). We used specific primers to identify resistant plants and wild-type (WT) plants as the negative control.

### 4.4. PCR Detection of Transgenic

In order to identify the integration of four genes (*GjsCCD4a*, *GjALDH2C3*, *GjUGT94E13*, *GjUGT74F8*) into the tobacco genome, the leaves of resistant plants’ and wild-type (WT) plants’ DNA was extracted using the Plant Genomic DNA Kit. Using plant genomic DNA as templates, a specific full-length primer was designed using 2× Rapid Taq Master Mix to amplify genes. All of the primers used in PCR detection are shown in Table S1.

### 4.5. Quantitative Detection of Gene Expression

RNA was extracted from wild-type and transgenic plants using CWBIO RNA extraction kit, followed by and reverse transcription; the first-strand cDNA was obtained from 1 μg of total RNA via the HiScript III 1st Strand cDNA Synthesis Kit (+gDNA wiper). The Taq Pro Universal SYBR qPCR Master Mix with ABI CFX96TM Real-Time System (Waltham, MA, USA) was used to detect the relative expression levels of *GjCCD4a*, *GjALDH2C3*, *GjUGT94E13*, *GjUGT74F8* and *Nbactin* as the internal control, WT plants as the negative control, and the expression levels of target genes were quantified via the 2^−ΔΔCT^ method. The data were presented as the mean values ± SDs of three independent experiments. The real-time PCR conditions were 95 °C for 30 s, then 40 cycles of 95 °C for 3 s and 55 °C for 10 s, and all the primers used for qRT-PCR are listed in Table S2.

### 4.6. Analysis of Crocins by HPLC-MS/MS

Crocins and crocetin extracted from 0.1 g transgenic tobacco leaves using 80% methanol via Ice bath ultrasound extraction for 1 h, centrifuged at 12,000× *g* for 10 min and then using 1 mL syringe absorbed the extract and filtered with HPLC filter tubes (0.45 mm). AB Sciex 4500 QTRAP LC/MS/MS (Toronto, ON, Canada) and an Agilent Technologies 1260 Series LC system (Agilent, Santa Clara, CA, USA) were used for HPLC-MS/MS analysis. Agilent Poroshell 120 SB C18 column (100 mm × 3.0 mm, 2.7 µm) was used for the detection of T0 generation and Agilent Poroshell 120 SB C18 column (100 mm × 2.1 mm, 2.7 µm) was used for detection of T1 generation. The column temperature was 45 °C and the mobile phase was 0.1% formic acid-water (A) and acetonitrile (B) with a flow rate of 0.3 mL/min. The HPLC condition of crocin analysis was as follows: 5% B for 0 min; 10% B for 1 min; 40% B for 5 min; 95% B for 2 min; keep 95% B for 2 min; 5% B for 10 s; 5% B for 4 min 10 s. This study used electrospray ionization (ESI) with multiple reaction monitoring (MRM) scanning, and the HPLC-MS/MS parameters are shown in Table 1. The characteristic ion pairs of each compound were extracted to obtain the peak areas. Each experiment was triplicated, and the quantitative analyses were performed using an external standard method. The standard samples were all trans configuration, with a purity of over 98%.

### 4.7. Determination of Moisture Content

The moisture content was determined using the drying method: 1–2 g of transgenic tobacco leaves were accurately weighed and dried in a constant weight weighing bottle at 105 °C for 6 h. They were transferred to a dryer for cooling for 30 min, accurately weighed, then dried at the same temperature for 1 h, cooled, and weighed until the difference between two consecutive times did not exceed 5 mg. Four parallel experiments were conducted.

### 4.8. Data Analysis

SPSS 16.0 statistics program (IBM Co., Armonk, NY, USA) was used to analyze the data in this study. All graphs were illustrated using Origin 2021 (OriginLab Co., Northampton, MA, USA) and MATLAB 2016a (MathWorks Co., Natick, MA, USA).

## Figures and Tables

**Table 1:** All compounds ion information.

**Analytes**	**Molecular Formula**	**Q1 Mass**	**Q2 Mass**	**DP (V)**	**CE (eV)**
Crocin I	976.7	999.3	675.0	120	50
Crocin II	814.8	837.3	675.0	120	50
Crocin III	652.7	675.3	351.2	120	45
Crocin IV	652.7	675.3	347.0	120	35
Crocin V	490.5	513.3	351.3	120	50
Crocetin	328.4	327.1	239.1; 119.2	−60	−20
**MS parameters**		**Crocetin**		**Crocins**	
Ion mode		Negative		Positive	
Source temperature (°C)		550		550	
Ionization voltage (V)		−4500		5500	
GS1 (psi)		55		55	
GS2 (psi)		55		55	
CUR (psi)		35		35	
CAD		Medium		Medium	
Dwell time (ms)		100		100	
EP (V)		−10		10	
CXP (V)		−15		15	

## Data Availability

The authors confirm that all data in this experiment are available in the main text and Supplementary data.

## Supplementary Materials

The following supporting information can be downloaded at https://www.mdpi.com/article/10.3390/ijms241814139/s1.
