# Supplementary material for: Synthesis of Crocin I and Crocin II by Multigene Stacking in Nicotiana benthamiana
Source: Int J Mol Sci. 2023 Sep 15;24(18):14139. doi: 10.3390/ijms241814139 (PMC10532124; doi:10.3390/ijms241814139)
Supplement: Supplementary file 1 [file ijms-24-14139-s001.zip › ijms-2583034-supplementary.pdf]

SUPPLEMENTARY INFORMATION

This file contains the Supplementary Figures and Supplementary Tables corresponding to the manuscript: **"Synthesis of crocin I and crocin II by multigene stacking** in Nicotiana benthamiana"

Lei Xie[1], Zuliang Luo[1], Xunli Jia[1], Changming Mo[2], Xiyang Huang[3], Yaran Suo [4], Shengrong Cui[1], Yimei Zang[1], Jingjing Liao[2*] & Xiaojun Ma[1*]

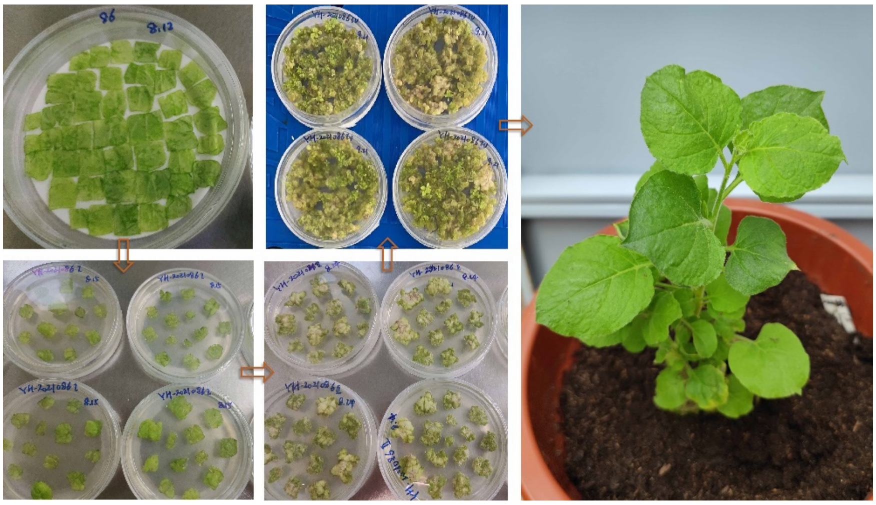

Figure S1. Process of agrobacterium tumefaciens-mediated transformation of multi-gene vector AU-CU into *Nicotiana benthamiana*.

TGAGACTTTTCAACAAAGGGTAATATCCGGAAACCTCCTCGGATTCCATTGCCCAGCTATCTGTC
ACTTTATTGTGAAGATAGTGGAAAAGGAAGGTGGCTCCTACAAATGCCATCATTGCGATAAAGG
AAAGGCCATCGTTGAAGATGCCTCTGCCGACAGTGGTCCCAAAGATGGACCCCCACCCACGAG
GAGCATCGTGGAAAAAGAAGACGTTCCAACCACGTCTTCAAAGCAAGTGGATTGATGTGATATC
TCCACTGACGTAAGGGATGACGCACAATCCCACTATCCTTCGCAAGACCCTTCCTCTATATAAG
GAAGTTCATTTCATTTGGAGAGAACAATGGCCGTCCAAAGCAACGGCAATGGAAGCTCGG
ATTCTCATGTTAAGGTTCCTCAGATAAAGTTCACCAAGCTCTTCATCAATGGAGAGTT
CGTTGATTCTGTTTCAGGAAAGACGTTTGAGACAATAGATCCAAGAAATGGGGAGGT
GATTGCAGAAATTGCCGAGGGGGGCAAGGAAGATGTTGATTTGGCTGTCAAAGCTGC
CCGAGAAGCTTTTGACCACGGCCCATGGCCTCGTTTACCTGGCTCTGAGAGGCGAAA
GATAATGATGAAGTTTGCAGACTTAATAGATGAAAATACCCAAGAATTGGCCACCTT
GGATGCAATTGATGCTGGAAAATTGTTTTACCTTTGCAAGATTATGGATATTCCAGGA
GCAGCAGAAACAATTCGCTATTATGCGGGTGCGGCAGATAAGATTCATGGGGAGACT
CTAAAAATGTCAACCGCGCTACAAGGATACACATTGCATGAGCCCATTGGAGTTGTT
GGACACATCATTCCCTGGAACTTCCCCAGTCAAATGTTTGCTATGAAGGTTGGCCCTG
CATTAGCAGCTGGCTGCACCATGGTAGTCAAGCCTGCTGAACAGACGCCTCTGTCAG
CCCTCTATTACGCTCATTTAGCAAAGCTGGCTGGAATCCCCGATGGAGTGCTAAACGT

TGTAACAGGATTCGGACAAACAGCTGGTGCGGCAATTAGCTCTCATATGGACATTGA
TATGGTAAGTTTCACGGGTTCTACAGAAGTAGGGCGTCTCGTCATGCAAGCTGCAGC
AACAAGCAACTTAAAGCCTGTGTGTCTAGAACTGGGAGGCAAGTCCCCCATTTTAAT
CTTCGATGATGCAGATGTTGACAAAGTTACAGAACTCGCACTGCAGGGAACTCTATTT
AACAAGGGTGAAATATGTGTTGCTGGCTCTCGTATTTTTGTTCAAGAGGGGATTTATG
ACAAGTTTTTAATCAAGTTGAAGCAGAAGGTGAAGAATTGGGTGGTCGGTGACCCTT
TTGATCCAACATCTCATCAAGGACCCCAAGTTGACAAGAAGCAGTACGATAGAATAC
TTTCATACATCGAGCATGGGAAGAAGGAAGGTGCCACCTTATTCCACGGTGGCAAGC
CTTGTGACGGGAAGGGCTACTACATTGAGCCAACAATATTCACTGATGTCACGGATG
AGATGACTATTGCGAAGGAAGAAATATTTGGGCCTGTGATGTCAGTATTCAAATTCA
AGACTGTTGAGGAGGCAATCAAGAGAGCAAATGCGACCAAATATGGACTGGCAGCG
GGCGTCATGACTAACAACATTAACATCGCCAACACCGTCGCAAGATCAATCCGTGCG
GGTGCTATCTGGATAAATTGTTACTTTGCCTTCGACAGAGATAGCCCTTACGGAGGCT
ACAAAATGAGTGGTTTCGGGAGAGACATGGGAATGGATGGCCTCAAGAAGTATCTTG
CAGTTAAAGCTGTTGCTACCCCCATTTATAATTCACCTTGGCTGGAGCAGAAACTCAT
CTCTGAAGAGGATCTGGGAAGCGGAGCTACTAACTTCAGCCTGCTGAAGCAGGCTGG
AGACGTGGAGGAGAACCCTGGACCTATGAAGGTACTAATGTTGCCATGGTTAGCTCA
TGGTCACATCTCTCCTTTCTTAGAGCTAGCCAAGAGGCTGGCAAAGAAAAACTTCCAC
ATCTACCTGTGTTCCACCTCTGTCAATCTCAGCTCCATCAAGAACAAGATTACCGGAG
AATATTCCGACTCAATAGAACCAGTTGAGCTACAGCTTCCATGCTTGCCTGATCTTCC
TCCTCATTACCACACCACCAATGGCCTCCCACCCCATCTCATGACCACCCTCAAAACA
GCCTATGAACTGTCAGCTCCCGACTTTTCTAATATCCTCACCGCTCTGCATCCTGACTT
GGTTGTGTACGACTTTAACCAGCCGTGGGCAGCAGAAATTGCTTCGTCTAAAAACATC
CCAGCAGTTCAATTTCTCCCGGTTGGGGCAACAATGATGGCTTTCAGTCTGCACATGC
TCAAGTATTCTGGAAAAGAATTCCCCCACCCAGAAATTTATATCCGGGACTACGAGA
TGCTCAAGTTTCAGTCAAGGAACGATCAAGTTAATGATGTTAGTGATAGAGAAAGAA
TCCTGCAAGCTCTGGACCTGTCGTGTAAGATTTTGTTGGTCAAGTCGTTCAAAGAAAT
CGAAGAAAAATTCATGAATACTCTCTCTGTTGCATCTGGTAAAAAGGTGGTCCCCGTT
GGCCCTCTTGTCCAAGACGTCAATATCGATGACATTCAAGATGAGGAAATGGAAATC
ATCCATTGGCTTGACCAAAAGGAAAATGCTTCAGTGGTGTTTGTCTCTTTTGGTAGTG
AGTATTTCTTGACTAAGGAGGAAAGAAACGAGATTGCTCGGGGTCTTGAGCTCAGCA
ATGTTAACTTTATATGGGTAATTAGGTTCCCTCTGGGAGGAAAAATAACTATGGAAGA
AGCATTGCCCGAAGGTTTTCTTGAGAGAGTGGGAGATAGAGGAAAAATTGTGGATGG
ATGGGCTCCTCAGGCAAGAATTTTGAAGCATGCGAATACTGGTGCTTTTTTGAGCCAT
TGTGGATGGAGTTCAATGATGGAGAGTATGAAATTTGGTGTTCCAATAATAGCTATGC
CTATGAGTGTTGATCAACCTGTGAATGCTAGATTGATTGAAGCAGTTGGAGTGGGATT
GGAGCCCTTGAGAGACGAGAAGGGGAATCTTCAAAGTGCGGAGATTGCCAAAGTGA
TTAGAAAGGTTTTGGTGGACGAAAGTGGAGAAAATGTGAGGAGGAAGGCTAAGGAA
TTGAGTGAACAAATGGAAATGAGAGGTGACGAGGAGGAGATTGATAACCTTGTAGA
GGAGCTGCTGCAACTCTGCCGAAAGAACAACGGGGGCTGTGATTACAAGGATGACGA
CGATAAGTAAATATGAAGATGAAGATGAAATATTTGGTGTGTCAAATAAAAAGGTTGTGTGCT
TAAGTTTGTGTTTTTTTCTTGGCTTGTTGTGTTATGAATTTGTGGCTTTTTCTAATATTAAATGAATG
TAACATCTCATTATAATGAATAAACAAATGTTTCTATAATCCATTGTGAATGTTTTGTTGGATCTCT
TCTCCAGCATATAACTACTGTATGTGCTATGGTATGGACTATGGAATATGATTAAAGATAAG

GTCGACGAGTCAGTAATAAACGGCGTCAAAGTGGTTGCAGCCGGCACACACGAGTCGTGT
TTATCAACTCAAAGCACAAATACTTTTCCTCAACCTAAAAATAAGGCAATTAGCCAAAAA
CAACTTTGCGTGTAAACAACGCTCAATACACGTGTCATTTTATTATTAGCTATTGCTTCACC
GCCTTAGCTTTCTCGTGACCTAGTCGTCCTCGTCTTTTCTTCTTCTTCTATAAAACAATA
CCCAAAGAGCTCTTCTTCTTCACAATTCAGATTTCAATTTCTCAAAATCTTAAAAACTTTCT
CTCAATTCTCTCTACCGTGATCAAGGTAAATTTCTGTGTTCCTTATTCTCTCAAAATCTTCG
ATTTTGTTTTCGTTCGATCCCAATTTCGTATATGTTCTTTGGTTTAGATTCTGTTAATCTTAG
ATCGAAGACGATTTTCTGGGTTTGATCGTTAGATATCATCTTAATTCTCGATTAGGGTTTCA
TAGATATCATCCGATTTGTTCAAATAATTTGAGTTTTGTCGAATAATTACTCTTCGATTTGT
GATTTCTATCTAGATCTGGTGTTAGTTTCTAGTTTGTGCGATCGAATTTGTCGATTAATCTG
AGTTTTTCTGATTAACAGATGGATGCTTTCTCTTCCTCTTTCCTCTCTCGACTATCCCAAA
ATCCCAAACTTCCGACTTCTCTAACCAACCCTTCATCACCAAATATTCCTTTACTACACA
TTTCATCCGCTAGAATTGAAGATAAGCCACAACCAACAATTATTACTAGCACCCCCATC
ACTGCCACAACGCCACCATCATCTAATTCTAATCCCCTTAAGAAGCAGAACCTACCGAA
ATATACACTTAAAAGATCCAATCCCAAGCCGCAATCATCATCACAACCACCACCATCAC
TGCAGCTGCAAGAACCTTCAAGAAAGCCAGTAGAACCATCATTGCTCACCAAATTCTT
CAATGCACTTGATAACTTCATAGACACATGCATTGATCCTCCAACTCACCCTTCTGTTGA
CCCCAAATACGTCCTGGCAAACAACTTTGTACCAGTTGATGAACTCCCCCCGACCGAA
TGTGAGGTGGTGGAAGGCTTTCTTCCACCGTGCCTTGACGGTGCCTACTTCCGCAATG
GCCCAAACCCTCAGTTCGTCACTCACGGGCCCTACCACTTTTTCGATGGCGATGGAATG
ATACATTCCATCAAAATCTCCGAAGGAAAAGCCACCTTCTGCAGCCGGTACGTGAAAA
CATACAAGTACATGACGGAACGGGATACGGGCTCTCCCGTGTTCCCTAAAGTATTCTCT
GGTTTCAATGGCCTAACAGCCTCCGCAGCCAGGGTTGCCCTAGCTGCTGCCAGAATGT
TAGCTGGCCAATTTAACCGAGCCAATGGTTTTGGCCTGGCAAACACTAGCTTGGCTCTA
GTTGGGGGGAAACTATTCGCTTTAGGTGAACCAGACCTTCCATATGCTGTAAAATTAAC
ACAAACTGGAGATTTAGTAACCGTAGGCCGTCATGACTTTGATGGTAAGCTTATTGCGA
GCATGACTGCACATCCCAAAATCGACCCTGAGACAGGTGAAGCTTTCGCATTCAGATA
CGCGCTAATGCCTCCATTTCTAACCTTCTTTAGAATCAACCCTGATGGTACAAAAGAAC
CAGACGTCCCGATTTTCTCCATGACACGGCCTTCATTTGTTCATGATTTGGCCATCACA
AGAAAATACGCTATTTTTCCTGACATACAAATAGGGATCAACCCTCTTCAGATGATCGC
CGGCGGACCACTAGTGGGTTCAGATCCCCGAAAAGTGCCACGACTCGGGGTTATTCCC
AGGTATGCTGCGGATGAAGCTGAGATGAAGTGGTTTGATGTTCCGGGACTTAATCTTAT
GCATGCTGTCAATGCTTGGGATGAAGATGATGGTGATGGTATTGTTGTTGTAGGAACTA
ATAGTTTGCCCATTGAGCATGTTCTTCGGCGGATGGATTTGGTTCATGCATCCATGGTGA
AAATCAAGATTGATCTCAAGACTGGGATGGTTTGGAAACATCCAATTTCTACGAGGAG
TATGGAATTTGGAGTCATTAATCCGGCTTATGTTGGCAAGAAGAACAAGTATGTATATGC
AGCAGTTGTAGACCCTTTGCCAAAGGTGTCAGGAGTGGTGAAGCTAGACTTATCAGCT
TCGGCAGGTGAGCACCGCGACTGCGTCATTGCTAGCCAGCTGTATGAACCGGAGTGCT
TTGGAAGCGAACCATTTTTCGTGGCCAAAGACCCTAACAACCCAAATGCAGATGAAGA
TGATGGTTATGTGGTGTCCTATGTCCATGATGAAAACACAGGAGAGTCAAGATTCTTAG
TGATGGACGCGAAGTCACCGAGTCTTGAAATTGTGGCTGCTGTAAAATTGCCTCAAAG
GGTTCCTTCTGGCTTTCATGGCCTCTTTGTCAGAGAAAGTGAGCTCAATAAATTGTACC
CATACGATGTTCCAGATTACGCTGGTTCAGGTGCCACTAATTTTTCTCTCTTGAAACAG
GCCGGTGACGTTGAAGAGAACCCAGGTCCAATGAATTCCAGCAAAGTTCATGTTTTAG

TTCTGCCATATCCTGCCCAGGGCCACATAAACCCCTTGCTCCAGTTCTGTAAACGTTTG
GTGGCAAAAGGTGTCAAAACCACGTTTGTCAACACTGTCTTCGTCTCCAAGTCCATTC
AACCAGATCCCAGCTGCTCAATTAACTTTGAGACCATTTCAGATGGCCATGACGAAGG
TGGCTATGCAGCAGCTGAAAGCCCAGAGGCCTATCTTGTAGAACTGGCCACCTTCGGC
TCAAAGACGTTGGCTGATCTTATCAGGAAGCTTGAAGACAAGGGCGAACCAGTCCAG
GCCGTGATTTATGATTCATTCCTGACATGGGCTCTTGATGTAGCTAAGCAATTTGGACTT
GTTACAGCTGCTTTTTTCACTCAGACTTGTGCTGTAAACAGCGTCTACTACCATGTTTAT
CATGGACTTCTACCGCTCCCTCTGTCAGACTCACCTCTTTCACTTCCTGGATTGCCGCT
GCTCCAACCCAAGGAAACTCCGTCCTTTGTTTACCTTCCCGATTCGTATCCTGCTTTTC
GTCTCATGCTCGTTAATCAGTTCTCCAACGTTGATCAAGCAGATTGGGTCATCCTCAAC
ATTTTCCACAAATTGGAGGAAGATGTGCTGAATTGGATAGCGAAACTTTGGCGAGTGA
TTACAGTCGGTCCAACGGTTCCATCCATGTACTTGGATAAACGCCTTGAGGACGATAAA
TGTTACGGAATAAATCTCTTCAAGCCAGACTCCTGCCTGTGCATGAACTGGCTAAATAA
TCAACCAAAGGACTCGGTAGTTTATGTATCATTCGGTAGCTGGACAGAAATTGACGTAA
AACAAGTGGAGGAAATAGCTTCAGCTTTGAAGGAAAGCGGATTCAAATTCTTGTGGGT
CGTGAGATCATTTGAGAAGGAAAAGCTTCCAAGCAACTTCGCTGAGGAGACGTCGGA
GAAAGGCTTGGTGGTGACATGGAGTCCACAGCTTGAGGTATTGGCACATGAATCCGTC
GCTTGTTTCGTCACTCATTGCGGGTTCAATTCAGTGCTGGAGGCGTTGAGTTTAGGGGT
GCCGGTGGTGGCCGCGCCGCAGTGGACGGACCAGCCCACCAACGCTAAGTTTCTGGA
GGATATTTGGGGAGTTGGTGTGAAGGCTGGTGCGGATGAGAAAGGAATTGTGACGAG
GGAAACGCTAGTTTCCTGCATAAGGGAGATTATGGAGGGAGAAAAAGGAAAGCAGAT
TAAAGAGAATGCCATTAAATGGAAGACTTTAGCCAAAGAAGCAGTTGATGAGGGCGG
AAGTTCTGATAAGAACATTGACGAATTTGTTGCAGAATTGGCTGGCCAGAAGGCCCAG
AACCATCATCACCATCACCATTAGACTAGTTCGAGCTTTCGTTCGTATCATCGGTTTCGACAA
CGTTCGTCAAGTTCAATGCATCAGTTTCATTGCGCACACACCAGAATCCTACTGAGTTTGAGTAT
TATGGCATTGGGAAAACTGTTTTTCTTGTACCATTTGTTGTGCTTGTAATTTACTGTGTTTTTTATTC
GGTTTTCGCTATCGAACTGTGAAATGGAAATGGATGGAGAAGAGTTAATGAATGATATGGTCCT
TTTGTTCATTCTCAAATTAATATTATTTGTTTTTTCTCTTATTTGTTGTGTGTTGAATTTGAAATTATA
AGAGATATGCAAACATTTTGTTTTGAGTAAAAATGTGTCAAATCGTGGCCTCTAATGACCGAAGT
TAATATGAGGAGTAAAACACTTGTAGTTGTACCATTATGCTTATTCACTAGGCAACAAATATATTT
TCAGACCTAGAAAAGCTGCAAATGTTACTGAATACAAGTATGTCCTCTTGTGTTTTAGACATTTAT
GAACTTTCCTTTATGTAATTTTCCAGAATCCTTGTCAGATTCTAATCATTGCTTTATAATTATAGTT
ATACTCATGGATTTGTAGTTGAGTATGAAAATATTTTTTAATGCATTTTATGACTTGCCAATTGATT
GACAACATGCATCAA

Figure S2. The nucleotide sequence of insertion fragment in the multigene vector AU-CU. The crocins biosynthesis genes *GjALDH2C3* (black), *GjUGT94E13* (blue), *GjCCD4a* (green), and *GjUGT74F8* (orange) were shown in this graph. P2A peptide sequences were highlighted in red. GjALDH2C3-2A-GjUGT9F13 was controlled by CaMV 35S promoter (highlighted in yellow), and the terminator was heat-shock protein 18.2 terminator (Thsp) (highlighted in light blue). GjCCD4a-2A-GjUGT74F8 was controlled by the AtUBQ10 promoter (highlighted in light green) and terminator from the pea rbcS-E9 gene (Te9) (highlighted in gray).

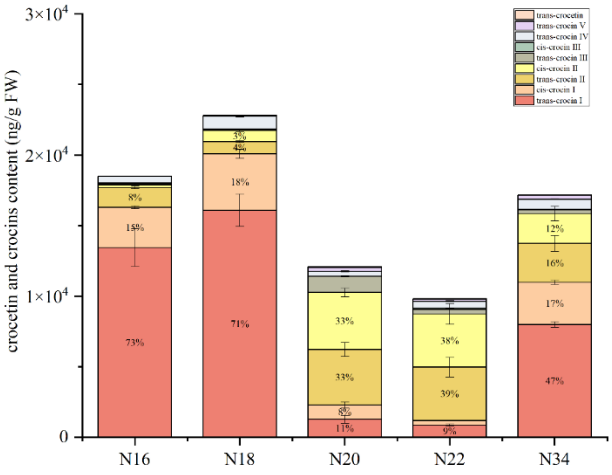

Figure S3. Crocetin and crocins production in transgenic tobacco of T1 generation lines. The data are presented as the mean values ± SDs, n = 3 biologically independent samples.

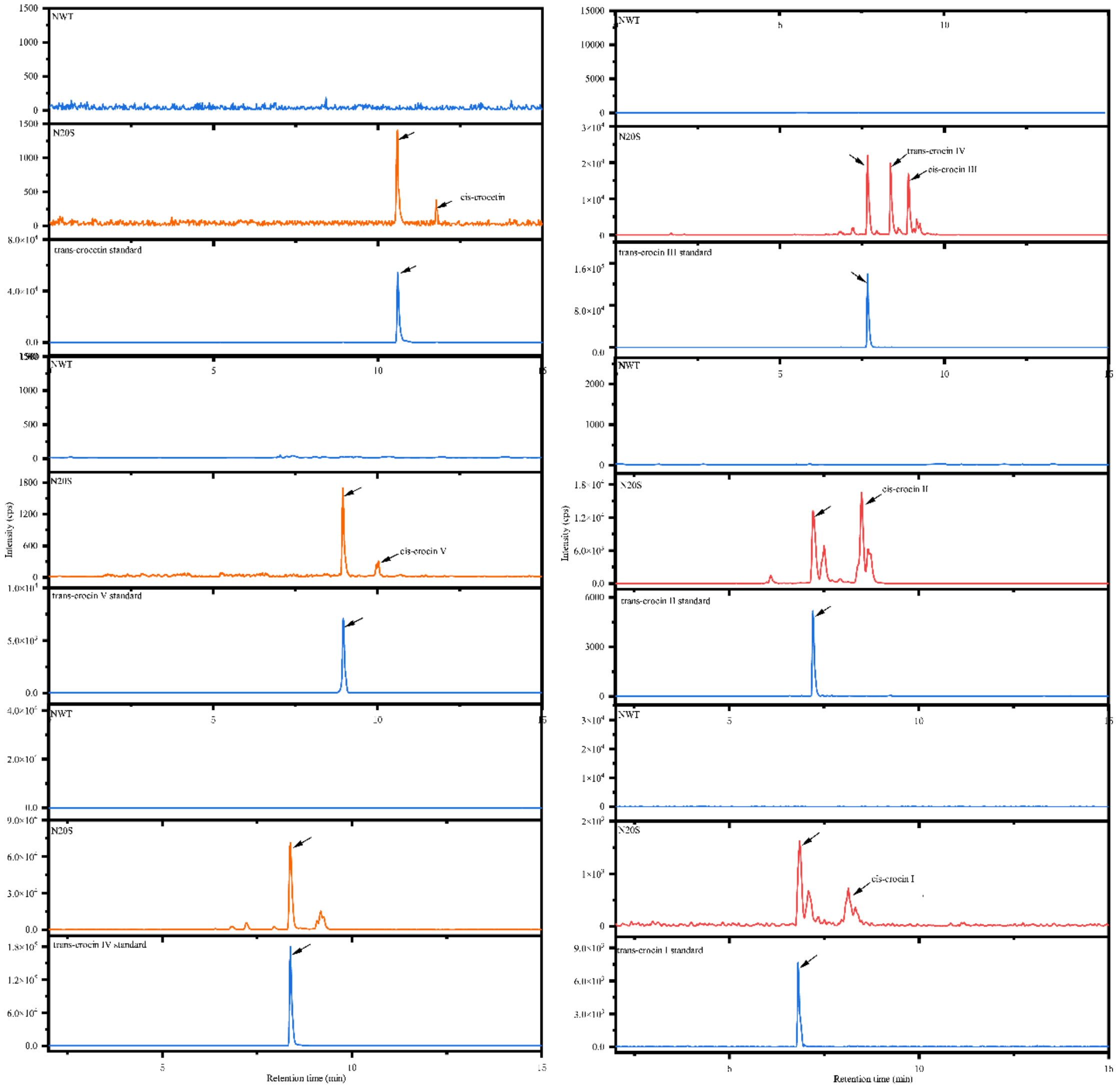

Figure S4. Identification of crocins in the stems of the transgenic tobacco N20 line.

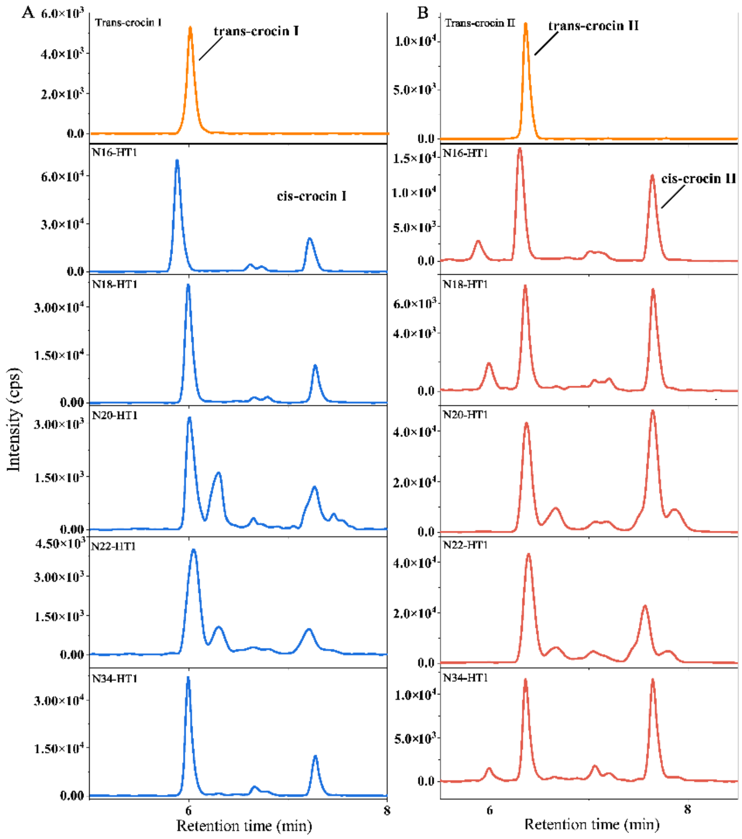

Figure S5. Analysis of crocin I and crocin II in the transgenic tobacco line N16-HT1, N18-HT1, N20-HT1, N22-HT1 and N34-HT1. (A) Ion pair 999.3/675.0 extraction chromatogram for analysis of crocin I in transgenic tobacco line. (B) Ion pair 837.3/675.0 extraction chromatogram for analysis of crocin II in transgenic tobacco line.

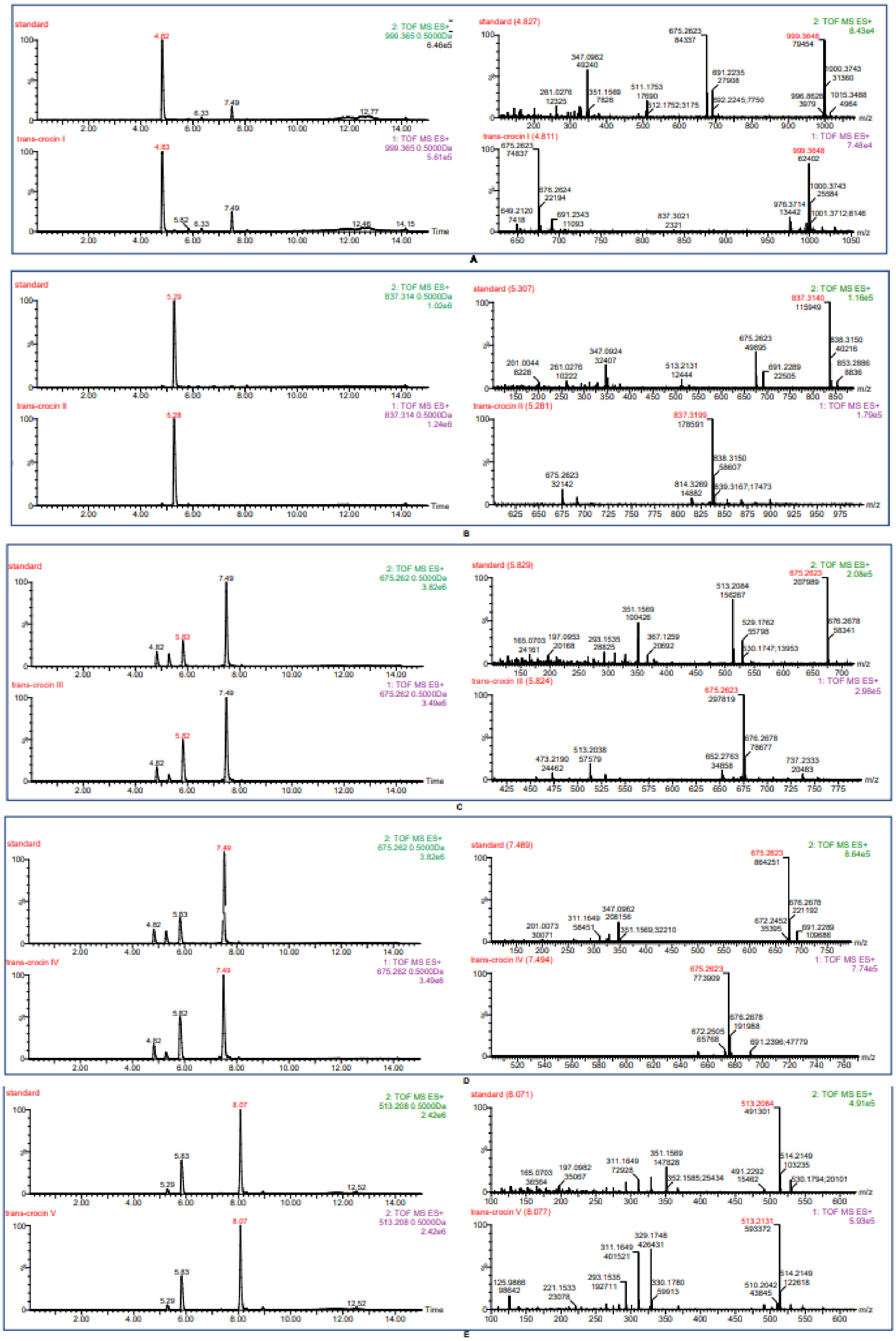

Figure S6. The UPLC-ESI-QTOF-MS/MS analysis of crocins for the standards. (A-E) The ion chromatogram, the MS and MS/MS spectra of crocin V-I.

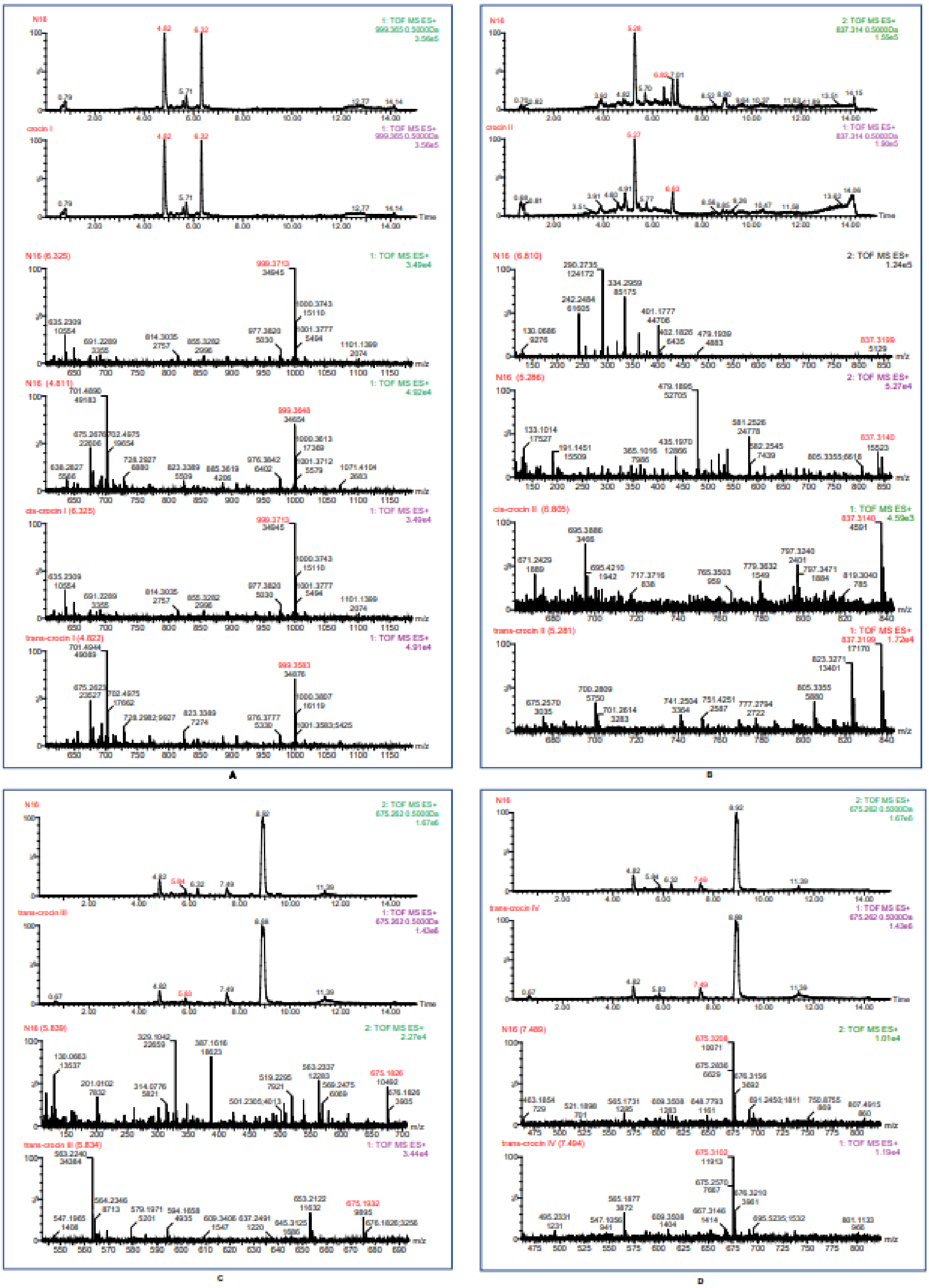

Figure S7. The UPLC-ESI-QTOF-MS/MS analysis of crocins for the N16 line. (A-D) The ion chromatogram, the MS and MS/MS spectra of crocin IV, crocin III, crocin II, and crocin I.

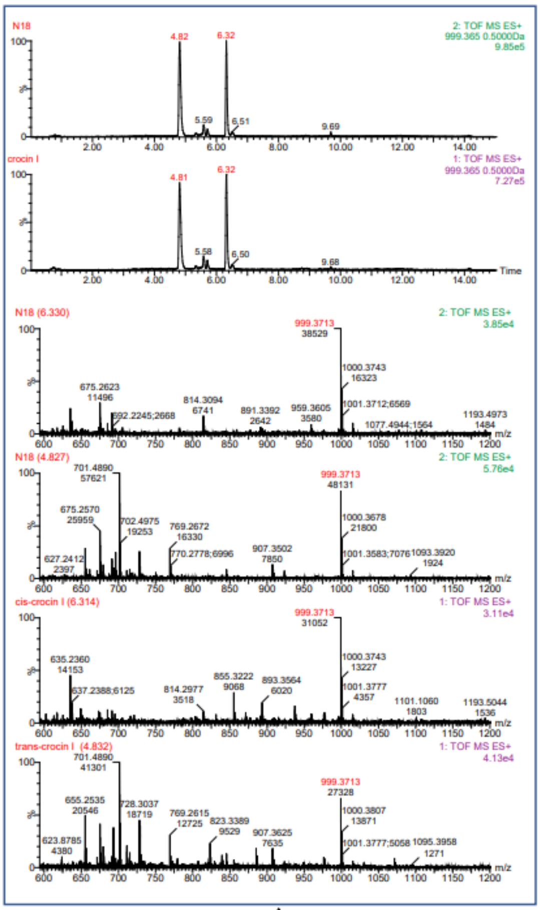
N18
crocin I
N18 (6.330)
N18 (4.827)
cis-crocin I (6.314)
trans-crocin I (4.832)

A

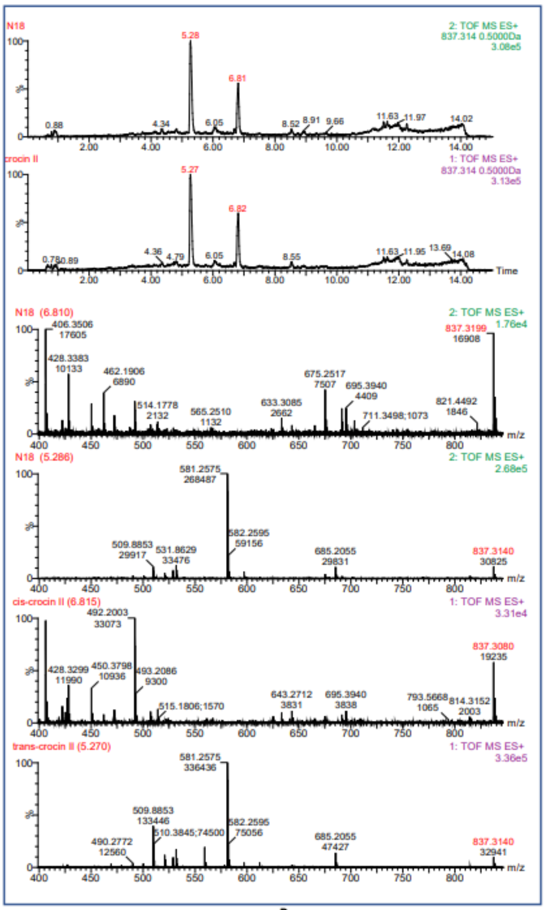
N18
crocin II
N18 (6.810)
N18 (5.286)
cis-crocin II (6.815)
trans-crocin II (5.270)

B

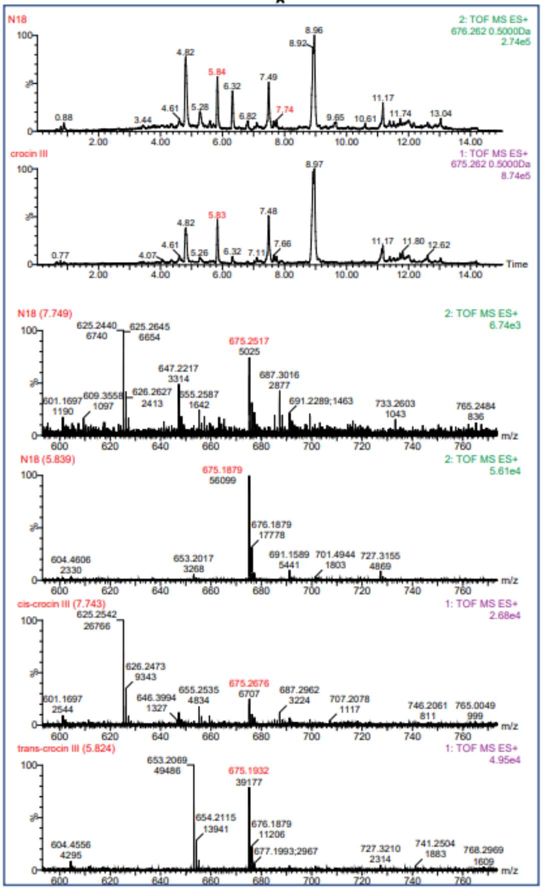
N18
crocin III
N18 (7.749)
N18 (5.839)
cis-crocin III (7.743)
trans-crocin III (5.824)

C

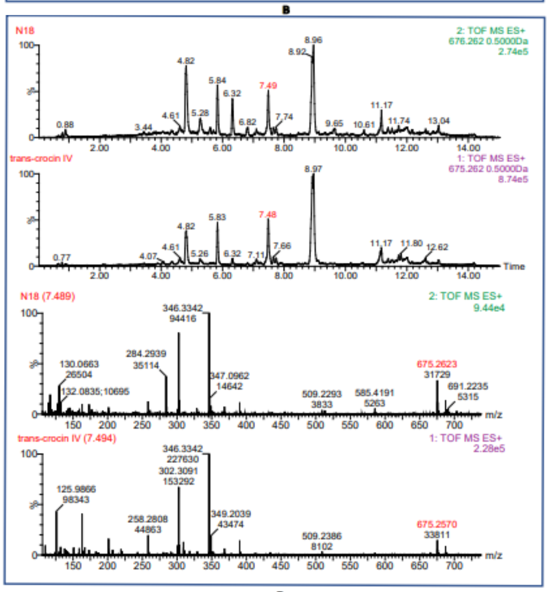
N18
trans-crocin IV
N18 (7.489)
trans-crocin IV (7.494)

D

Figure S8. The UPLC-ESI-QTOF-MS/MS analysis of crocins for the N18 line. (A-D) The ion chromatogram, the MS and MS/MS spectra of crocin IV, crocin III, crocin II, and crocin I.

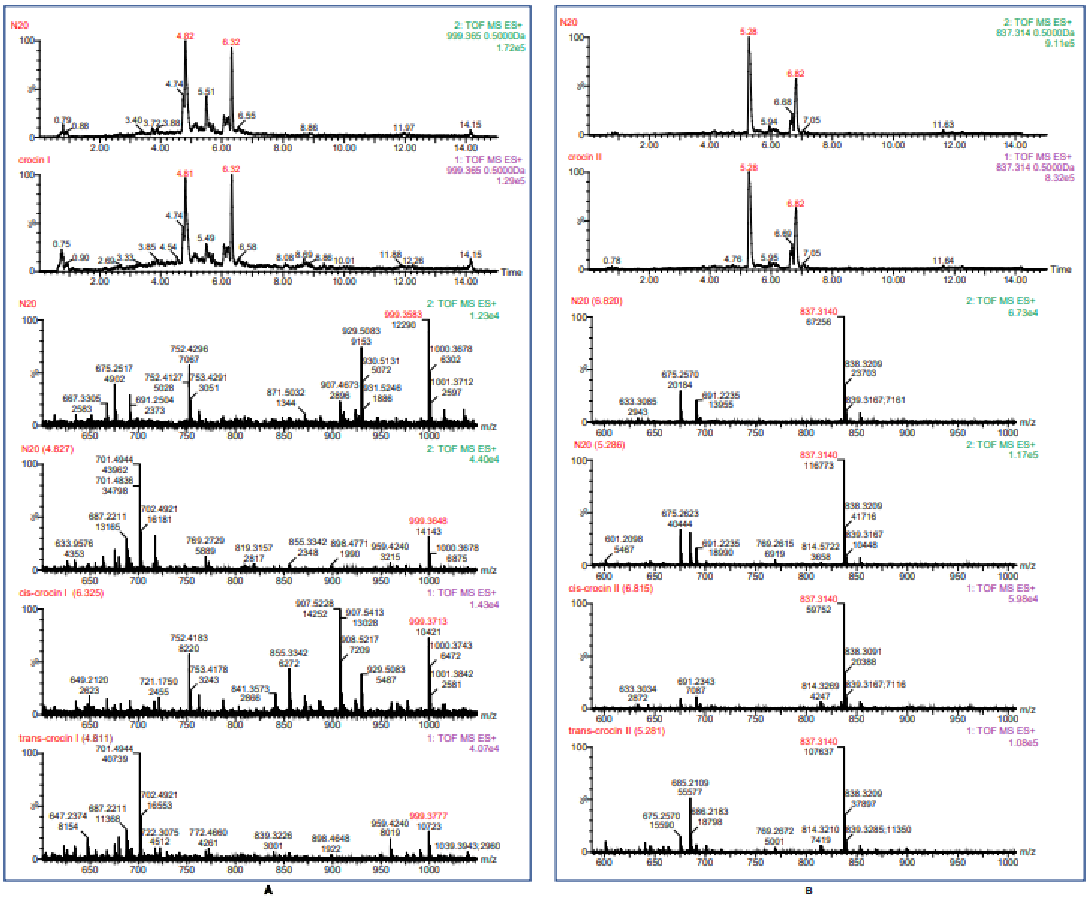

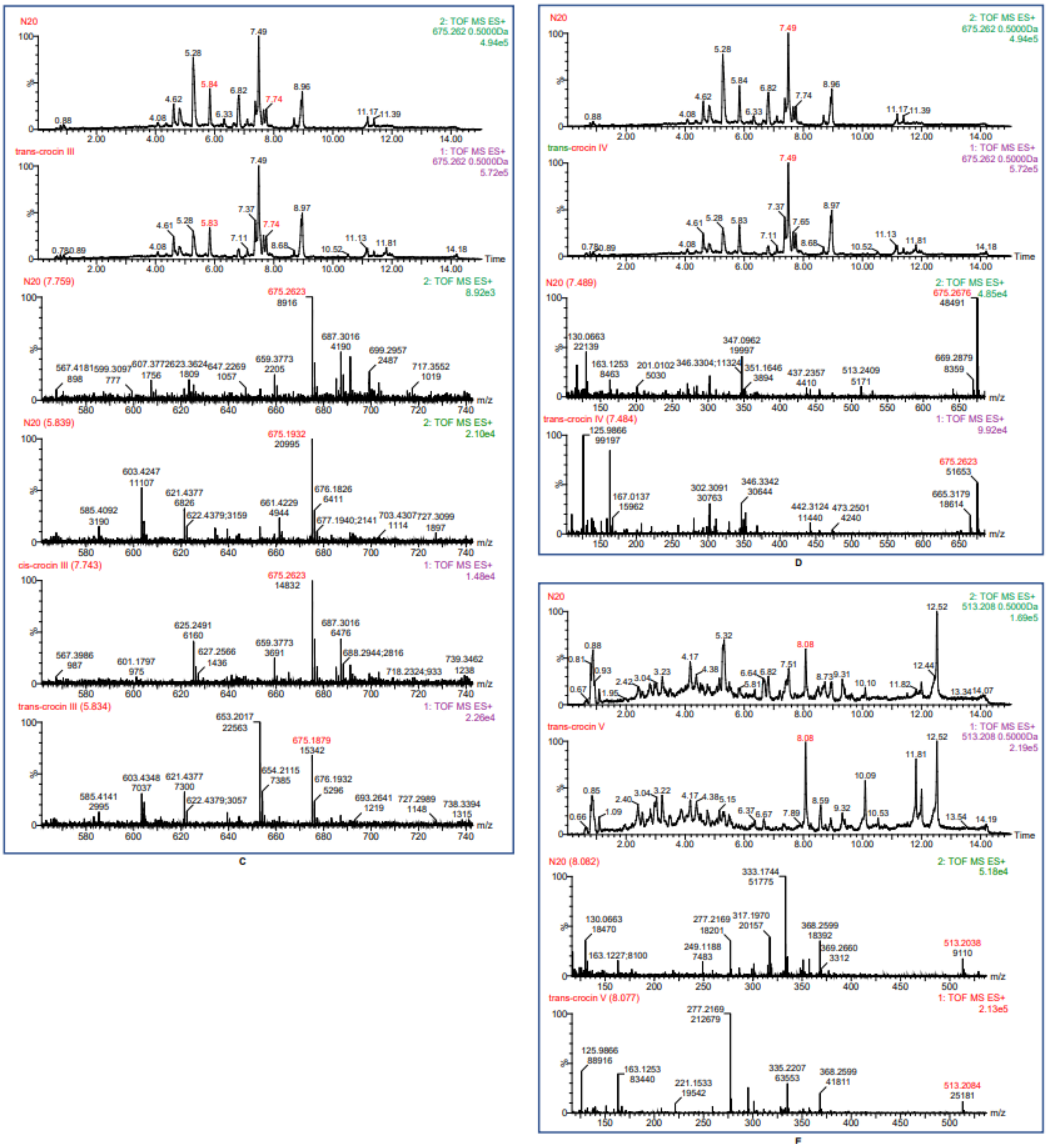

Figure S9. The UPLC-ESI-QTOF-MS/MS analysis of crocins for the N20 line. (A-E) The ion chromatogram, the MS and MS/MS spectra of crocin V, crocin IV, crocin III, crocin II, and crocin I.

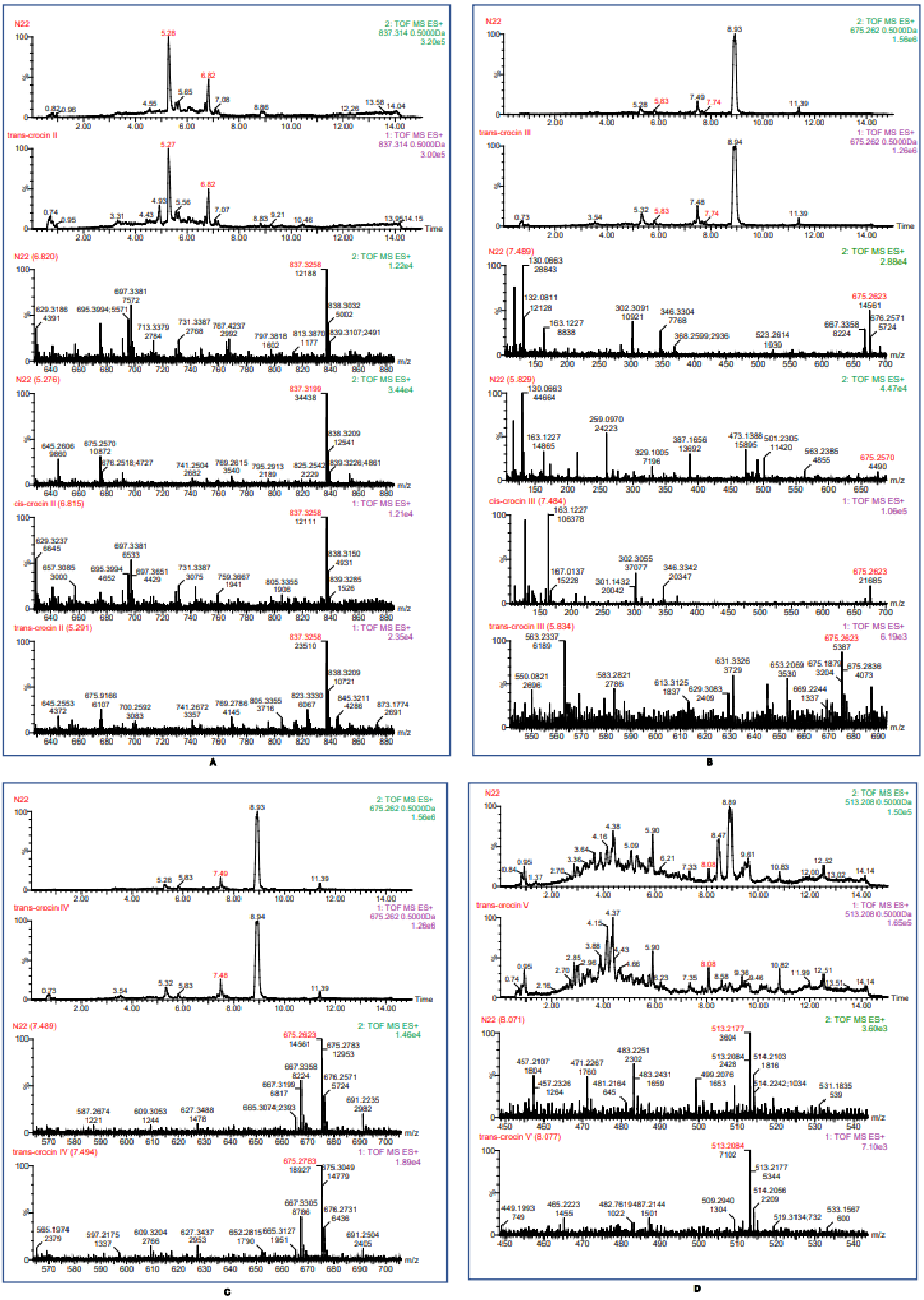

Figure S10. The UPLC-ESI-QTOF-MS/MS analysis of crocins for the N22 line. (A-D) The ion chromatogram, the MS and MS/MS spectra of crocin V, crocin IV, crocin III, and crocin I.

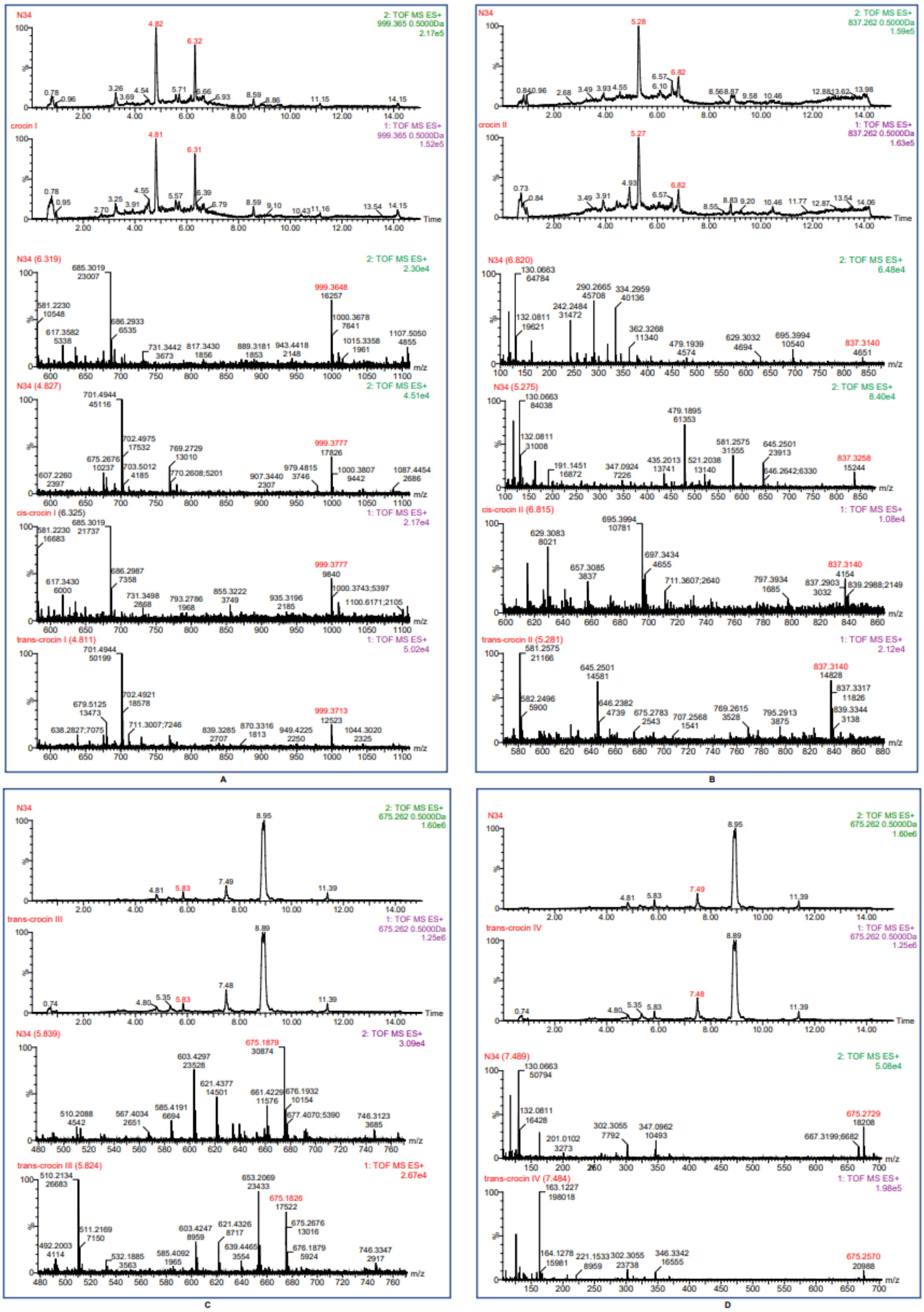

Figure S11. The UPLC-ESI-QTOF-MS/MS analysis of crocins for the N34 line. (A-D) The ion chromatogram, the MS and MS/MS spectra of crocin IV, crocin III, crocin II, and crocin I.

Primers	Sequences (5' to 3')
***GjCCD4a*-F**	ATGACTGATTTATTGTGTTTCCTATACG
***GjCCD4a*-R**	TTACAATTTATTGAGCTCACTTTCTCTG
***GjALDH2C3*-F**	ATGGCCGTCCAAAGCAACG
***GjALDH2C3*-R**	TTACAGCCAAGGTGAATTATAAATGG
***GjUGT74F8*-F**	ATGAATTCCAGCAAAGTTCATGTT
***GjUGT74F8*-R**	CTAGTTCTGGGCCTTCTGGCC
***GjUGT94E13*-F**	ATGAAGGTACTAATGTTGCCATGG
***GjUGT94E13*-R**	TTAACAGCCCCCGTTGTTCTT

Table S1. Primers for PCR detection

Primers	Sequences (5' to 3')
***GjCCD4a*-qF**	CAAGATTGATCTCAAGACTGG
***GjCCD4a*-qR**	GTCTACAACTGCTGCATATAC
***GjALDH2C3*-qF**	GGATTCTCATGTTAAGGTTCC
***GjALDH2C3*-qR**	GGATCTATTGTCTCAAACGTC
***GjUGT94E13*-qF**	CAATAGAACCAGTTGAGCTAC
***GjUGT94E13*qR**	GATATTAGAAAAGTCGGGAGC
***GjUGT74F8*-qF**	CAAAGGACTCGGTAGTTTATG
***GjUGT74F8*-qR**	CTTCTCAAATGATCTCACGAC
***NbActin*-qF**	TATTCCCTAGTATTGTTGGC
***NbActin*-qR**	CTGGGGTATTAAAAGTCTCA

Table S2. Primers for qRT-PC
